# Combined endophytic inoculants enhance nickel phytoextraction from serpentine soil in the hyperaccumulator *Noccaea caerulescens*

**DOI:** 10.3389/fpls.2015.00638

**Published:** 2015-08-14

**Authors:** Giovanna Visioli, Teofilo Vamerali, Monica Mattarozzi, Lucia Dramis, Anna M. Sanangelantoni

**Affiliations:** ^1^Department of Life Sciences, University of ParmaParma, Italy; ^2^Department of Agronomy, Food, Natural Resources, Animals and the Environment, University of PadovaPadova, Italy; ^3^Department of Chemistry, University of ParmaParma, Italy

**Keywords:** plant growth-promoting endophytic bacteria (PGPE), serpentine soil, nickel, tissue colonization, phytoextraction, *Noccaea caerulescens*, *Arabidopsis thaliana*

## Abstract

This study assesses the effects of specific bacterial endophytes on the phytoextraction capacity of the Ni-hyperaccumulator *Noccaea caerulescens*, spontaneously growing in a serpentine soil environment. Five metal-tolerant endophytes had already been selected for their high Ni tolerance (6 mM) and plant growth promoting ability. Here we demonstrate that individual bacterial inoculation is ineffective in enhancing Ni translocation and growth of *N. caerulescens* in serpentine soil, except for specific strains Ncr-1 and Ncr-8, belonging to the *Arthrobacter* and *Microbacterium* genera, which showed the highest indole acetic acid production and 1-aminocyclopropane-1-carboxylic acid-deaminase activity. Ncr-1 and Ncr-8 co-inoculation was even more efficient in promoting plant growth, soil Ni removal, and translocation of Ni, together with that of Fe, Co, and Cu. Bacteria of both strains densely colonized the root surfaces and intercellular spaces of leaf epidermal tissue. These two bacterial strains also turned out to stimulate root length, shoot biomass, and Ni uptake in *Arabidopsis thaliana* grown in MS agar medium supplemented with Ni. It is concluded that adaptation of *N. caerulescens* in highly Ni-contaminated serpentine soil can be enhanced by an integrated community of bacterial endophytes rather than by single strains; of the former, *Arthrobacter* and *Microbacterium* may be useful candidates for future phytoremediation trials in multiple metal-contaminated sites, with possible extension to non-hyperaccumulator plants.

## Introduction

Phytoextraction has been proposed as a low-cost effective technology for remediation of contaminated soils and for phytomining (Chaney et al., 2007). Phytoextraction implies the cultivation of plants which can accumulate trace metals from contaminated soils and transport them to the above-ground biomass, and then be harvested. Metal hyperaccumulator plants are ideal candidates for this technology, thanks to their extraordinary capacity for absorbing and accumulating metals in their shoots without showing symptoms of phytotoxicity (Baker et al., 2000). Unfortunately, most hyperaccumulators do not produce substantial quantities of biomass, thus hampering the management of phytoextraction. Understanding of the mechanisms underlying hyperaccumulation may allow this technology to be transferred to metal-tolerant and high biomass-yielding plants (Ebbs and Kochian, 1998).

In recent years, attention has focused on microorganisms thriving in the rhizosphere or inhabiting the roots of hyperaccumulators which show plant growth-promoting activity (Sessitsch et al., 2013; Visioli et al., 2015). Plant growth-promoting rhizobacteria (PGPR) and endophytes [Plant growth-promoting endophytic bacteria (PGPE)] can also enhance plant tolerance, growth, and survival in stress conditions of metal-rich soils by reducing the nutrient deficiency and phytotoxicity of trace metals. This is achieved by the production of siderophores, carboxylic acids, and solubilisation of phosphates, which increase the mobility of macro- and micronutrients in the rhizosphere (Rajkumar et al., 2012; Bashan et al., 2013a,b). PGPR and PGPE can also promote growth, both indirectly by protecting plants against pathogens and directly by producing phytohormones such as indole acetic acid (IAA), abscisic acid, and gibberellic acid, and by secreting enzymes such as 1-aminocyclopropane-1-carboxylic acid (ACC) deaminase, which inhibits ethylene production and consequently slows down plant aging. By lowering ethylene levels in plant tissues, ACC-deaminase reduces plant stress and improves growth under metal contamination, this being the main beneficial effect in phytoremediation (Kamnev, 2003; Spaepen et al., 2007; Belimov et al., 2009). The positive effects of PGPR and PGPE on plant growth and metal bioavailability can markedly improve metal accumulation by roots and shoots, thus efficiently influencing phytoremediation of metal-polluted sites (Li et al., 2007; Ma et al., 2009a,b, 2011a; Rajkumar et al., 2009, 2012; Chen et al., 2010; Sessitsch et al., 2013).

In many cases, the effects of plant–microbe interactions on growth and metal uptake has been shown to be species- and soil-specific (Sheng et al., 2008; Becerra-Castro et al., 2012; Cabello-Conejo et al., 2014), but interesting results have also been obtained by inoculating Ni-resistant PGPR isolated from the rhizosphere of *Alyssum serpyllifolium* and *Astragalus incanus* on the growth and Ni accumulation of *Ricinus communis*, *Heliantus annus*, and *Brassica juncea*, grown in single and multiple metal-contaminated soils (Ma et al., 2011a,b, 2015a; Jing et al., 2014). Cd- and Pb-mobilizing rhizospheric bacteria also enhanced the uptake of metals in tomato plants (Jiang et al., 2008) and Zn-mobilizing bacteria, isolated from serpentine soils, promoted Zn, Cu, and Ni accumulation in *R. communis* (Rajkumar and Freitas, 2008). Recent studies have further confirmed the potential role of PGPE in metal accumulation. For instance, endophytes from the *Bacillus* genus isolated from the roots of the Zn/Cd hyperaccumulator *Sedum plumbizincicola*, can effectively enhance plant biomass and Cd or Zn uptake (Ma et al., 2015b). A Cd-mobilizing endophytic strain isolated from maize roots can also improve Cd uptake by hyperaccumulator plants of the genus *Amaranthus* (Yuan et al., 2014); *Rahnella* sp. JN6, isolated from *Polygonum pubescens*, can also promote growth and Cd, Pb, and Zn uptake in the biomass species *B. napus* (He et al., 2013).

The increasing number of bacterial strains with beneficial effects on plant growth and metal accumulation traits, isolated from contaminated soil environments, both in the rhizosphere and from the roots of hyperaccumulator plant species, may contribute to the creation of a “bacteria phytoremediation database,” to be used to check the effectiveness of bacterial inocula on various high-yielding biomass plant species, for possible future applications in the phytomanagement of polluted sites.

Within this framework, we studied the interaction between the Ni-hyperaccumulator *Noccaea caerulescens* and five PGPE, previously isolated from its roots in a serpentine soil. Bacteria were supplied as the seed inoculum of each strain, both separately and in combination, in order to identify any synergistic mechanism. Characterisation of IAA production and ACC- deaminase activity in these bacteria also showed which of these physiological traits are efficient selection criteria for improving assisted metal phytoextraction. In this study, we documented plant-bacteria interactions by *in vivo* and *in vitro* microscopic observations, and attempted to transfer the technology *in vitro* to the non-hyperaccumulator *Arabidopsis thaliana* with the most efficient bacterial isolates.

## Materials and Methods

### Bacterial Strains

The endophytic bacteria tested here, Ncr-1, Ncr-3, Ncr-5, Ncr-8, and Ncr-9, had been isolated in a previous study from inners of the roots of *N. caerulescens* plants collected in serpentine soil in the Northern Italian Apennines (Visioli et al., 2014). They were selected from ten different strains on the basis of their greater ability to tolerate high concentrations of Ni in hydroponics (6 mM) and to produce plant growth-promoting substances (**Table 1**).

**Table 1:** Enzymatic and hormonal activity (mean ±SD, *n* = 3) of bacterial strains isolated from roots of Ni-hyperaccumulator *N. caerulescens* in a previous work (Visioli et al., 2014).

Bacterial strain	Accession number	Closest described relative	Ni resistance			Siderophore production	IAA production (mg L^-1)^	ACC deaminase activity (μM αKB mg^-1^ h^-1^)
			1 mM	3 mM	6 mM			
Ncr-1	KJ92857	*Arthrobacter sp.*	+++	+++	+++	+	25.6 ± 1.3	20 ± 2.5
Ncr-3	KJ92859	*Kocuria rhizophila*	+++	+++	+++	+	14.5 ± 2.8	ND
Ncr-5	KJ92861	*Bacillus sp.*	+++	+++	+++	+	10.6 ± 3.6	11.01 ± 2.0
Ncr-8	KJ92864	*Microbacterium oxydans*	+++	+++	+++	+	20.8 ± 2.4	30 ± 2.9
Ncr-9	KJ92865	*Bacillus amyloliquefaciens*	+++	+++	+++	+	11.3 ± 0.8	ND

*ND, not detected; ACC, 1-aminocyclopropane-1-carboxylic acid; αKB, α-ketobutyrate; IAA, indole-3-acetic acid.*

### Bacterial Seed Inoculation and Plant Germination *In Vitro*

Ncr-1, Ncr-3, Ncr-5, Ncr-8, and Ncr-9 strains were grown overnight in 100 mL Luria Bertani (LB) medium at 30°C on a rotary shaker. Cells were collected by centrifugation and suspended in LB medium to obtain a final inoculum density of 10^8^ CFU mL^-1^. *N. caerulescens* seeds were surface sterilized in 50% (v/v) commercial bleach (5% sodium hypochlorite and 0.05% sodium hydroxide) in water for 15 min and then rinsed for 5 min in sterile water three times. Seeds were kept for 2 h in a bacterial suspension of 5 × 10^8^ cells mL^-1^ of each strain or in a bacterial suspension of both Ncr-1 and Ncr-8 strains (5 × 10^8^ cells mL^-1^ of each) and then thoroughly washed in sterilized water. The seeds were then plated on 1× MS (Murashige and Skoog, 1962) agar medium and incubated in a vertical position in an environmentally controlled room (22°C; 16 h/8 h light/dark; 120 μmol m^-2^ s^-1^ PAR, 75% RH) to determine germination and root elongation. The same sterilization and co-inoculation procedures were used for *A. thaliana* seeds with Ncr-1 and Ncr-8 strains.

### Soil Experiments

The soil used in these experiments was collected at a previously characterized site on Mount Prinzera, a serpentine outcrop in the Northern Italian Apennines (GPS 44.64282°N-10.07951°E; for basic soil properties, see Visioli et al., 2013, and **Table 2**).

**Table 2:** Physicochemical characteristics of serpentine soil used for growing inoculated and control *N. caerulescens* plants.

Parameters
pH	7.2-7.5
Organic matter (%)	2.95 ± 0.21
Water content (%)	19.01 ± 0.2
Ca/Mg	0.25 ± 0.0
**Total metal concentration (mg kg**^-^**^**1**^)**
Fe	47120 ± 341 (62.5 ± 3.4)
Ni	1244 ± 24 (34.8 ± 3.4)
Zn	71 ± 0.2 (1.4 ± 0.01)
Co	97 ± 1.3 (7.3 ± 0.03)
Cu	11 ± 0.1 (2.3 ± 0.01)

*Diethylene triamine pentaacetic acid (DTPA)-extractable fractions are reported in parenthesis.*

Soil pH was measured with a glass electrode from a deionised water suspension (20 g soil/50 mL water) after 1 h agitation and overnight settlement. Organic matter was determined on soils sampled at depths of 10 and 20 cm as LOI (weight loss on ignition; Storer, 1984). Aliquots of 2 g of each sample were placed in closed ceramic crucibles for 3 h at 450°C; organic matter was calculated as the fraction of weight decrease. Soil water content was measured on 20-g samples, placed in closed heat-resistant plastic containers, previously weighed and placed in an oven at 70°C for 24 h. The percentage of water in the sediment was calculated as weight loss from the initial weight. The soil was then sieved (2 mm) and sterilized at 80°C for 24 h.

Fourteen-day-old *N. caerulescens* seedlings (three per pot), inoculated and non-inoculated with the various bacterial strains, were transferred into PVC pots (upper diameter 110 mm, lower diameter 90 mm, height 100 mm) containing 750 g of soil, with three replicates. The plants were grown in the same conditions as above and the soil was wetted with sterile water and maintained at 25% w/w of water content (corresponding to 50% of its holding capacity). The experiment was set up in September 2014 and lasted 60 days, after which the plants were carefully lifted from the pots and the soil was removed from their roots. Plant roots were immersed in a 10 mM EDTA solution for 30 min and rinsed thoroughly with deionised water to avoid any metal contamination. Shoots and roots were then dried for 3 days at 70°C and root and shoot dry weights (DW) were recorded.

Hundred-mg samples of dried shoots or roots were microwave-acid digested (Milestone ETHOS 900, Bergamo, Italy) by the addition of 7 mL ultrapure grade 6 HNO_3_ (65% v/v) and 1 mL suprapur H_2_O_2_ (30% v/v), according to the EPA 3052 method (USEPA, 1995). The microwave setting reached 200°C in 10 min (step 1), followed by 10 min at 200°C (step 2), and a final air cooling phase down to <30°C. In steps 1 and 2, maximum pressure and power were 45 bar and 1.2 kW, respectively. Samples were then diluted to 25 mL with distilled water, filtered (0.45 μm PTFE) and analyzed by ICP-OES (SPECTRO Ciros Vision EOP, Spectro Analytical Instruments, Kleve, Germany) to reveal metal concentrations. Certified reference materials (ERM-CD281 and BRC-402, JRC-IRMM, Belgium) were used to ensure measurement accuracy. Data are expressed in mg kg^-1^ DW plant material. The Ni translocation factor (TF) was measured as the shoot-to-root metal concentration ratio.

Aliquots of 300 mg of soil collected in the rhizosphere of both inoculated and non-inoculated plants were also dried overnight at 70°C and wet-ashed; total Ni concentration was determined by ICP-OES, with the same procedure as above.

In addition, in order to evaluate Ni, Zn, Cu, and Co bioavailability, the same samples underwent diethylene triamine pentaacetic acid (DTPA) extraction. Bioavailable metals were extracted on 50 g of homogenized air-dried soil through a 100-mL solution of DTPA (1.97 g L^-1^), calcium chloride bihydrate (1.46 g L^-1^), and triethanolamine (14.92 g L^-1^), pH 7.3, shaken for 2 h (60 cycles min^-1^). Samples were analyzed by ICP-OES after centrifugation (5 min, 2599 × *g*).

The ability of Ncr-1, Ncr-3, Ncr-5, Ncr-8, and Ncr-9 to bioconcentrate Ni in the above-ground biomass of *N. caerulescens* from serpentine soil (BCF: Bioconcentration factor) was calculated as the ratio between shoot Ni concentration and soil pseudo-total Ni concentration. The effect of microbial inoculation on overall Ni phytoextraction efficiency was assessed by taking into account plant growth, and was calculated as the product of DW shoot yield and its Ni concentration.

After log-transformation of the response variables, ANOVA and Tukey’s *post hoc* test were used to ascertain differences in root length, root and shoot biomass and metal concentrations.

### Electron Microscopy Analysis

Fresh roots from 14-day-old *N. caerulescens* seedlings inoculated with Ncr-1 and Ncr-8 were directly collected from the plates and rinsed briefly in sterile water; 5-mm root sections were then excised with a sterile lancet. Unfixed and hydrated samples were directly analyzed under an Environmental Scanning Electron Microscope (ESEM) Quanta^TM^ 250 FEG (FEI, Hillsboro, OR, USA), operating in wet mode conditions. In more detail, samples were placed on double-sided adhesive carbon tape fixed to a pre-cooled metal sample holder, in thermal contact with a Peltier cooling stage maintained at 3°C. Accelerating voltage was 10 kV and the secondary electron signal was collected by a gaseous secondary electron detector (GSED) to generate micrographs for morphological studies. Relative humidity was initially set at 100%, and then slowly decreased to 80% by adjusting chamber pressure.

### Optical Microscopy Analysis

Five-mm root and leaf sections were excised with a sterile lancet from *in vitro* 14-day-old *N. caerulescens* seedlings, inoculated and non-inoculated with Ncr-1 and Ncr-8 strains, collected from Petri dishes, and immediately fixed overnight at 4°C in formalin-acetic acid-alcohol (FAA). After this step, samples were dehydrated in a tertiary butyl-alcohol series and gradually embedded in paraffin. They were then cut into sections 5 μm thick with a rotary microtome (Reichert-Jung 2040) and stained with safranin-fast green (Berlyn and Miksche, 1976). Lastly, they were analyzed under a Nikon eclipse E600 microscope mounted with a DS-FIZ camera.

## Results and Discussion

### Plant Biomass Yield

Solving the problem of interactions between *N. caerulescens* and PGPE in serpentine soil, which is characterized by low levels of essential nutrients and elevated Ni, together with other toxic metals, can be illuminating in understanding plant–microorganism interactions in an extremely adverse environment and the potential use of metal-resistant endophytic bacteria in phytomanagement of metal-polluted sites.

In a previous work, five culturable bacterial endophytes were selected among 10 isolates from *N. caerulescens* roots according to their high resistance to Ni and ability to produce PGP metabolites such as IAA, ACC-deaminase, and siderophores (**Table 1**).

These five inoculants promoted plant growth and Ni translocation in a hydroponic system, enriched with 10 μM Ni and supplied adequately with nutrients for growth (Visioli et al., 2014). Since in the real environment soil properties can greatly affect shoot growth and bacterial root colonization, essential nutrients often being limiting factors, in the present study the performance of these bacteria were further investigated in conditions more similar to those of open fields. The serpentine soil used was the original soil from which the Ni-hyperaccumulator *N. caerulescens* was first collected. The soil has a relatively low content of organic matter (<3%) and neutral pH (**Table 2**). In addition, it has a low Ca/Mg ratio, responsible for Ca uptake inhibition, and high levels of Mg (>300 mg kg^-1^; Visioli et al., 2013) and several toxic metals, particularly Ni (>1000 mg kg^-1^). Low levels of available macronutrients N, K, P were further edaphic constraints which severely affect plant growth and reproduction.

**FIGURE 1:**
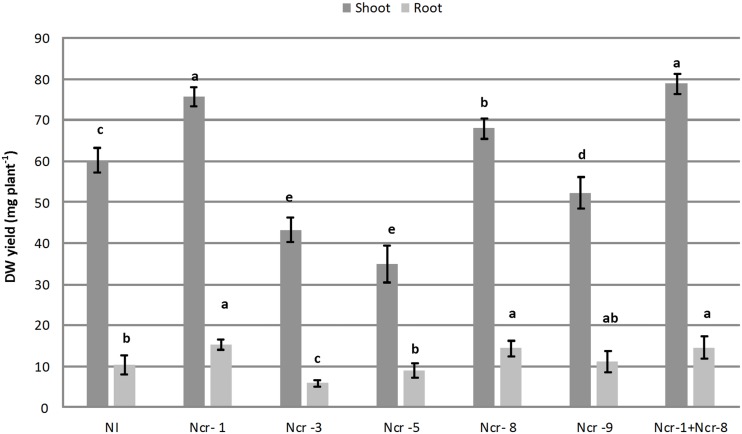
**Effects of five endophytic inoculants and co-inoculation of Ncr-1 + Ncr-8 PGPE strains compared with non-inoculated plants (NI) on shoot and root biomass (mg plant^-1^) of *N. caerulescens* growing on serpentine soil in pot experiment.** Data of each plant batch (three replicates) derived from three plants. Values with different letters are significantly different among treatments (*p* < 0.05).

*Noccaea caerulescens* seeds were either inoculated with the five bacterial strains separately, or co-inoculated with strains Ncr-1 and Ncr-8 together, in order to determine the potential additive effects of PGPE on plant growth and Ni accumulation in serpentine soil. Both Ncr-1 and Ncr-8 showed the highest IAA production and AAC-deaminase activity (**Table 1**). Among treatments with single inoculants, only plants treated with Ncr-1 and Ncr-8 showed significant higher shoot biomass compared with untreated controls, i.e., +25 and +12%, respectively (**Figure 1**). Co-inoculum with Ncr-1 and Ncr-8 had a synergistic effect, with a further shoot DW increase of 4 and 16% with respect to Ncr-1 and Ncr-8 alone, respectively. Plant inoculation with Ncr-3, Ncr-5, and Ncr-9 produced a significant decrease in shoot and seldom (Ncr-3) in root biomasses, compared with non-inoculated controls, suggesting their poor interaction with *N. caerulescens* (**Figure 1**). The beneficial effects of bacterial inoculants on the growth of metal-exposed plants have often been attributed to the production and transfer to plants of high IAA levels (Spaepen et al., 2007; Dell’Amico et al., 2008). The positive influence of the Ncr-1 + Ncr-8 combination on the biomass yield may be due to their substantial release of auxin, associated with reduced ethylene production through increased ACC-deaminase activity. IAA increases plant growth by promoting cell division or stimulating cell elongation (Spaepen et al., 2007), whereas ACC-deaminase effectively reduces ethylene production by plants, retarding leaf senescence (Glick et al., 1998) and increasing plant yield (Belimov et al., 2009).

The data presented here are consistent with recent results from the literature, evidencing the positive effect on growth of the Ni-hyperaccumulator *Alyssum pintodasilvae* of PGPR and PGPE strains belonging to *Arthrobacter* and *Microbacterium* genera in a serpentine soil (Cabello-Conejo et al., 2014). The beneficial role of PGPE belonging to the *Bacillus* genus on the growth of the Cd/Zn hyperaccumulator *S. plumbizincicola* grown in a multiple-metal contaminated soil has also recently been demonstrated (Ma et al., 2015b).

Among bacterial inoculants, plant growth impairment was particularly evident with strain Ncr-3, since both shoot and root biomass were reduced by 28 and 39%, respectively, compared with non-inoculated plants. Ncr-3 showed great similarity to *Kocuria rhizophila*, and its negative effect on *N. caerulescens* is in contrast with results in recent literature, which reports a positive influence on growth and chromium accumulation in *Cicer arietinum* L, although with a different *K. flava* species isolated from the rhizosphere of chickpea (Singh et al., 2014). Our *Kocuria* strain, coming from the rhizosphere of *N. caerulescens*, was found to have poor IAA production, and lacked ACC-deaminase activity, probably indicating the importance of this hormone and enzymatic activities in establishing positive interactions with plants.

### Ni Uptake and Translocation, and Shoot and Root Ionome

**FIGURE 2:**
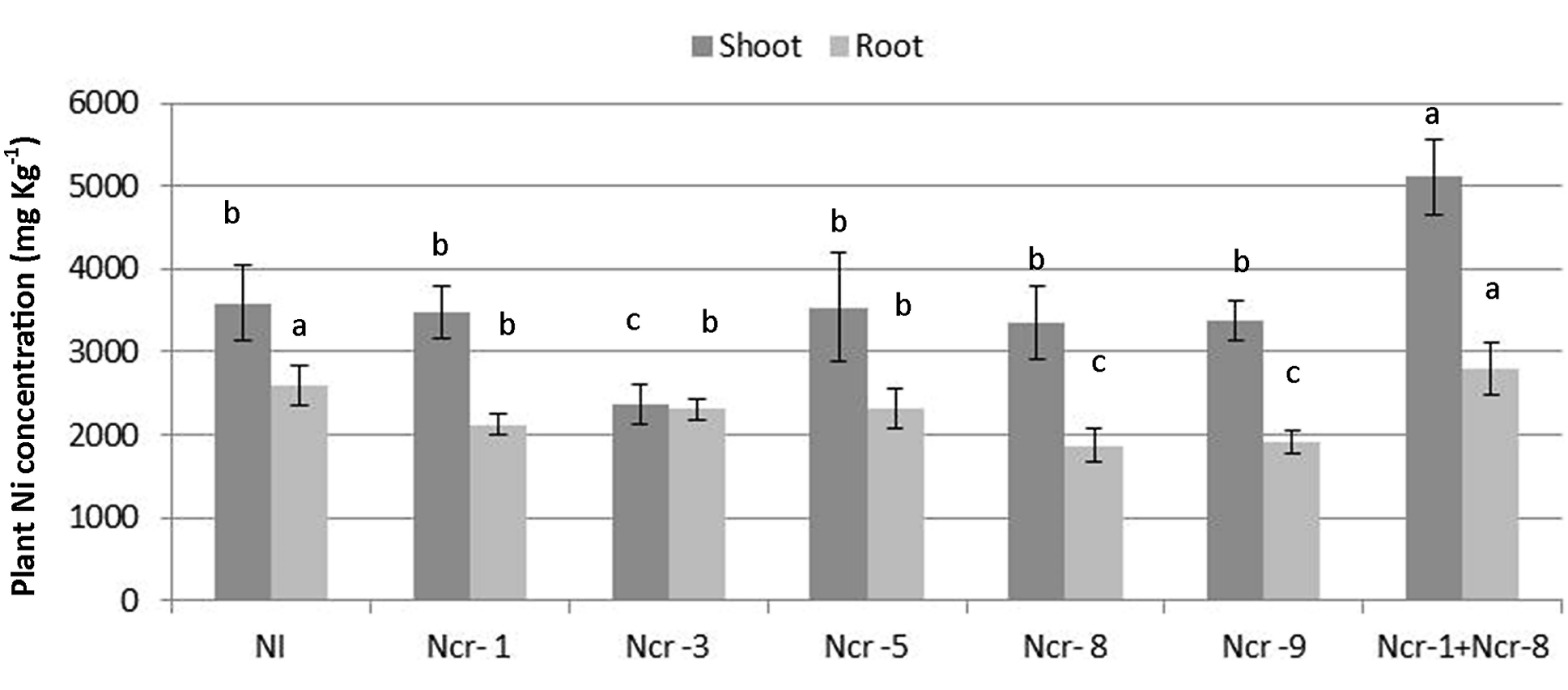
**Effects of five endophytic inoculants and co-inoculation of Ncr-1 + Ncr-8 PGPE strains compared with non-inoculated plants (NI) on shoot and root Ni concentration (mg kg^-1^ DW) of *N. caerulescens* on serpentine soil in pot experiment.** Data of each plant batch (three replicates) derived from three plants. Values with different letters are significantly different among treatments (*p* < 0.05).

The Ni rate in roots of Ncr-1 + Ncr-8 co-inoculated plants and untreated controls was very similar (2.6 ± 0.4 g kg^-1^ vs. 2.7 ± 0.4 g kg^-1^), although significantly lower values (*p* < 0.05) were observed in all other treatments with single strains. Analysis of shoot tissues confirmed that Ni was significantly higher in the co-inoculated NCr-1 + NCr-8 plants compared with untreated controls (5.1 ± 0.3 g kg^-1^ vs. 3.5 ± 0.2 g kg^-1^), and the absence of significant differences among control plants and single-strain inoculated plants, with the exception of Ncr-3, which showed reduced rates (2.3 ± 0.1 g kg^-1^; **Figure 2**). Anyway, shoot Ni rates in *N. caerulescens* were extremely high with all bacteria strains, according with the capability of this Ni-hyperaccumulator species. The shoot-to-root Ni ratio was >1 in all treatments except for Ncr-3 (TF = 1.0; **Table 3**) and was improved with strains Ncr-1 and Ncr-8 and their co-inoculation, confirming the general ability of *N. caerulescens* to accumulate Ni above-ground and involvement of these bacteria in Ni uptake and translocation (**Table 3**).

**Table 3:** Nickel translocation factor (TF), calculated as shoot-to-root Ni concentration ratio and bioconcentration factor (BCF), determined as ratio of shoot Ni and total soil Ni concentrations (mean ±SD, *n* = 3) in *N. caerulescens* inoculated with five bacterial strains.

	NI	Ncr-1	Ncr-3	Ncr-5	Ncr-8	Ncr-9	Ncr-1 + Ncr-8
TF	1.4	1.6	1.0	1.5	1.8	1.8	1.8
BCF	2.9 ± 0.1	2.7 ± 0.5	1.9 ± 0.2	2.8 ± 0.3	2.7 ± 0.2	2.7 ± 0.4	4.0 ± 0.5^∗^

*^∗^Significant differences (p < 0.05) between non-inoculated (NI) and treated plants.*

Nevertheless, Ni soil bioavailability was similar before and after plant growth and did not apparently change in the rhizosphere as a consequence of bacteria inoculation, with an average value of 35.4 mg kg^-1^ as DTPA extractable fraction at end of the experiment (**Table 4**). Compared with non-inoculated controls, inoculants led to some small changes only in Ni phytoavailability, with an increase with Ncr-8 and Ncr1 + Ncr8 co-inoculation. The same effect was observed for Zn, Co, and Cu, with no variations following inoculation.

**Table 4:** Diethylene triamine pentaacetic acid- extractable fractions of metals (mean ±SD, *n* = 3) in rhizosphere soil of inoculated and control *N. caerulescens* plants at harvest (end of experiment).

	Ni	Zn	Co	Cu
	mg kg^-1^	mg kg^-1^	mg kg^-1^	mg kg^-1^
NI	34.8 ± 3.4	1.4 ± 0.01	7.3 ± 0.03	2.3 ± 0.01
Ncr-1	35.4 ± 2.5	1.4 ± 0.03	7.4 ± 0.02	2.3 ± 0.02
Ncr-3	34.8 ± 1.3	1.5 ± 0.05	7.3 ± 0.04	2.4 ± 0.03
Ncr-5	34.3 ± 1.2	1.7 ± 0.06	7.3 ± 0.05	2.3 ± 0.07
Ncr-8	36.5 ± 2.9^∗^	1.4 ± 0.10	7.1 ± 0.10	2.4 ± 0.02
Ncr-9	35.8 ± 2.5	1.5 ± 0.05	7.1 ± 0.02	2.2 ± 0.01
Ncr1 + Ncr8	36.2 ± 1.3^∗^	1.5 ± 0.04	7.0 ± 0.01	2.3 ± 0.01

*^∗^Significant difference (p < 0.05) between inoculated plants and non-inoculated (NI) controls.*

In the literature, contrasting results are described as regards the effects of rhizosphere bacteria on soil metal mobility. For instance, *Microbacterium arabinogalactolyticum* was found to increase the soil extractability of Ni (Abou-Shanab et al., 2003, 2006), although no variations were observed with *Arthrobacter nitroguajacolicus* and *Microbacterium* sp. inoculants in serpentine soil with the Ni-hyperaccumulator *A. pintodasilvae* (Cabello-Conejo et al., 2014). The dynamic nature of metal solution-solid phase interactions would explain the absence of a direct correlation between Ni uptake and its DTPA extractable fraction.

**FIGURE 3:**
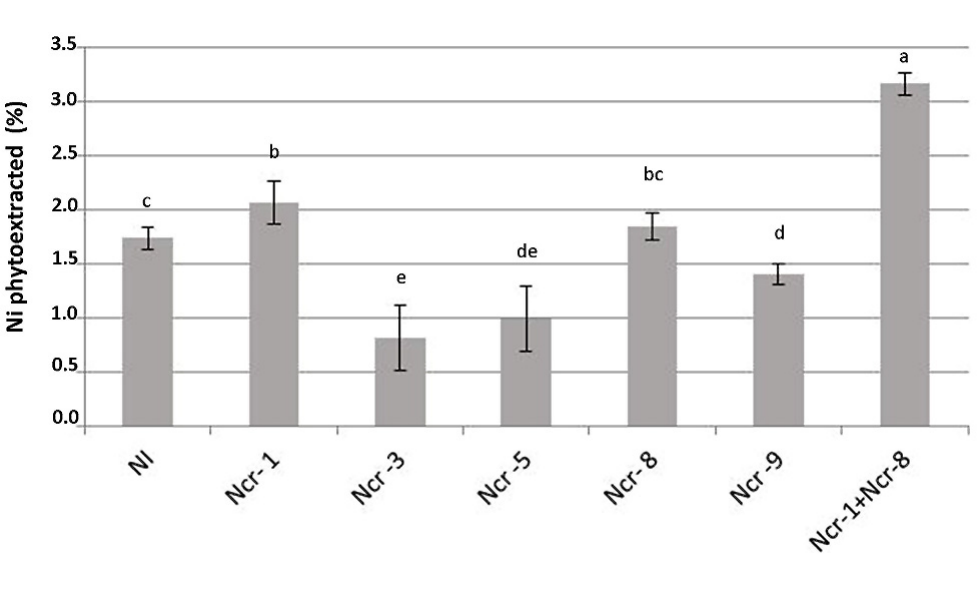
**Effects of five endophytic inoculants and co-inoculation of Ncr-1 + Ncr-8 PGPE strains compared with non-inoculated plants (NI) on Ni phytoextraction (% of shoot Ni removed from total soil Ni) by *N. caerulescens* growing on serpentine soil in pot experiment.** Data of each plant batch (three replicates) derived from three plants. Values with different letters are significantly different among treatments (*p* < 0.05).

Co-inoculation of Ncr-1 and Ncr-8 strains appreciably improved the BCF, i.e., 1.38 times higher than that of untreated controls (*P* < 0.05; **Table 3**). As a consequence, the percentage of Ni removal was significantly higher than in non-inoculated plants (3.2% vs 1.7%); in single-strain inoculated plants, only Ncr-1 showed significantly higher Ni phytoextraction (2.1%; **Figure 3**). Ncr-8 led to the same phytoextraction capacity as controls, whereas Ncr-3, Ncr-5, and Ncr-9 had significantly lower capacities. These results indicate that, with the combined help of two selected bacteria, *N. caerulescens* can take up Ni from the soil more efficiently than in aseptic conditions, confirming previous findings on the stimulation effects of endophytes on metal uptake in higher plants (He et al., 2013; Yuan et al., 2014; Ma et al., 2015b).

As regards other elements, both shoot and root tissues had similar concentrations of the main macronutrients Ca, K, Mg, P, and S between inoculated and untreated plants. The only exception was Ncr-3, which showed a significant decrease in shoot K, together with root K, S, and Mg increases (**Table 5**). Some trace metals also seldom changed between treatments; bacterial inoculation generally led to a substantial Zn decrease in shoots, regardless of bacterial strain, and a generalized Cu decrease in roots, except for Ncr-3 (**Table 5**). The response of Co was peculiar, in that distribution in both shoot and roots only increased with Ncr-1 + Ncr-8 co-inoculation.

**Table 5:** Mean macro- and micro-nutrient concentrations (expressed in g kg^-1^) in shoots and roots of *N. caerulescens* grown in serpentine soil with five bacterial strains inoculants.

	Ca	K	P	S	Mg	Fe	Zn	Mn	Co	Cu

						g kg^-1^
**Shoots**					
NI	14.3 ± 2.1	58.7 ± 4.5	3.8 ± 0.33	6.0 ± 1.13	11.7 ± 2.0	0.86 ± 0.11	0.58 ± 0.10	0.38 ± 0.02	0.15 ± 0.01	0.013 ± 0.02
Ncr-1	18.7 ± 7.7	55.1 ± 3.4	3.4 ± 0.45	5.5 ± 0.90	13 ± 1.5	1.37 ± 0.53^∗^	0.34 ± 0.08^∗^	0.35 ± 0.05	0.18 ± 0.02	0.014 ± 0.03
Ncr-3	14.8 ± 3.2	38.8 ± 4.8^∗^	2.7 ± 0.25	4.7 ± 0.34	17 ± 3.6	1.04 ± 0.24^∗^	0.16 ± 0.05^∗^	0.30 ± 0.03	0.13 ± 0.01	0.017 ± 0.02^∗^
Ncr-5	15.8 ± 2.3	48.3 ± 2.3	3.8 ± 0.12	6.4 ± 1.43	16.3 ± 2.8	1.26 ± 0.37^∗^	0.23 ± 0.04^∗^	0.34 ± 0.02	0.17 ± 0.04	0.009 ± 0.01^∗^
Ncr-8	14.2 ± 2.5	52.0 ± 2.2	3.2 ± 0.50	5.2 ± 0.87	12 ± 1.8	1.50 ± 0.22^∗^	0.28 ± 0.03^∗^	0.35 ± 0.03	0.16 ± 0.02	0.016 ± 0.02
Ncr-9	15.3 ± 5.1	51.1 ± 2.8	3.1 ± 0.23	5.1 ± 1.65	12.6 ± 3.0	0.71 ± 0.70	0.28 ± 0.02^∗^	0.32 ± 0.04	0.16 ± 0.02	0.017 ± 0.04^∗^
Ncr1 + Ncr8	16.4 ± 3.2	47.6 ± 3.4	2.5 ± 0.15	6.3 ± 0.34	12.2 ± 2.5	0.80 ± 0.27	0.25 ± 0.04^∗^	1.18 ± 0.06^∗^	0.67 ± 0.02^∗^	0.014 ± 0.03
**Roots**					
NI	7.3 ± 0.9	80.3 ± 3.5	4.5 ± 0.26	7.9 ± 0.23	14.8 ± 2.0	2.8 ± 0.1	0.33 ± 0.05	0.45 ± 0.3	0.038 ± 0.01	0.059 ± 0.01
Ncr-1	8.2 ± 1.2	68.9 ± 6.3	6.0 ± 0.37	9.4 ± 0.45	15.0 ± 2.4	3.0 ± 0.3	0.41 ± 0.04	0.56 ± 0.2	0.060 ± 0.02	0.034 ± 0.02^∗^
Ncr-3	8.8 ± 0.2	127.0 ± 8.0^∗^	5.4 ± 0.25	12.6 ± 1.32^∗^	19.0 ± 1.7^∗^	3.5 ± 0.2	0.35 ± 0.031	0.78 ± 0.2^∗^	0.066 ± 0.02	0.060 ± 0.05
Ncr-5	9.4 ± 1.3	76.8 ± 2.5	4.8 ± 0.10	8.6 ± 0.28	22.3 ± 3.5	5.4 ± 0.3	0.54 ± 0.02	0.84 ± 0.01^∗^	0.088 ± 0.01^∗^	0.021 ± 0.001^∗^
Ncr-8	7.6 ± 0.4	74.7 ± 4.0	4.5 ± 0.31	8.2 ± 0.45	15.3 ± 2.6	3.8 ± 0.4	0.38 ± 0.01	0.44 ± 0.01	0.064 ± 0.01	0.028 ± 0.02^∗^
Ncr-9	6.7 ± 0.5	91.6 ± 3.2	4.6 ± 0.65	8.5 ± 0.32	15.4 ± 1.1	3.7 ± 0.2	0.27 ± 0.01	0.56 ± 0.05	0.051 ± 0.02	0.031 ± 0.02^∗^
Ncr1 + Ncr8	8.2 ± 1.6	89.4 ± 2.8	4.0 ± 0.19	10.5 ± 1.50	13.7 ± 2.1	0.5 ± 0.1	0.32 ± 0.05	0.95 ± 0.03^∗^	0.077 ± 0.07^∗^	0.020 ± 0.001^∗^

*^∗^Significant difference (p < 0.05) between inoculated plants and non-inoculated (NI) controls.*

As a consequence of such variations in plant metal rates, a general decrease in the shoot-to-root concentration ratio for Zn (i.e., TF) was observed in inoculated plants, whereas only co-inoculation with Ncr-1 and Ncr-8 significantly increased the TF of Fe, Co, and Cu compared with untreated controls and single inoculants (**Table 6**). According to some recent studies, siderophore production and P solubilisation by rhizosphere microrganisms play important roles in increasing the mobility of several trace metals in polluted substrates, thus facilitating their accumulation in plant tissues (Sessitsch et al., 2013). In our case, both Ncr-1 and Ncr-8 had good siderophore production, but real improvements in Ni, Mn, and Co accumulation in the above-ground biomass of *N. caerulescens* were observed when the ability of the two efficient strains were combined. As suggested by Rajkumar et al. (2012), siderophores can chelate the unavailable ferric form of iron in near-neutral pH conditions, allowing more efficient uptake of this metal by *N. caerulescens* roots, and we recorded a slight increase in shoot and root Fe accumulation with some bacterial strains. Instead, the general reduction in shoot Zn of all inoculated treatments was probably due to the mobilization of various metals present at higher concentrations in serpentine soils. These soils are commonly poor in Zn (∼70 mg kg^-1^ DW in our case) and competition with other abundant metals such as Ni (>1000 mg kg^-1^ soil DW), Co (>90 mg kg^-1^ soil DW) and Cu may explain the lower uptake and translocation of Zn. This hypothesis was apparently not supported by the stable DTPA-extractable fraction of the most abundant trace metals, but quantification of metal mobility at harvest is a final result which may not have described specific variations during the developmental stages of plants.

**Table 6:** Shoot-to-root concentration ratio (TF) of main macro- and micro-nutrients (mean ± SD, *n* = 3) in *N. caerulescens* grown in serpentine soil with five bacterial strain inoculants.

	Ca	K	P	S	Mg	Fe	Zn	Mn	Co	Cu
	
						TF
NI	2.0	0.7	0.8	0.8	0.8	0.3	1.8	0.8	3.9	0.2
Ncr-1	2.3	0.8	0.6	0.6	0.9	0.4	0.8^∗^	0.6	3.0	0.4
Ncr-3	1.7	0.3	0.5	0.4	0.9	0.3	0.5^∗^	0.4	2.0	0.2
Ncr-5	1.7	0.6	0.8	0.7	0.7	0.2	0.4^∗^	0.4	1.9	0.4
Ncr-8	1.9	0.7	0.6	0.6	0.8	0.4	0.7^∗^	0.8	2.5	0.6
Ncr-9	2.3	0.6	0.7	0.6	0.8	0.2	1.1	0.6	3.1	0.6
Ncr1 + Ncr8	2.0	0.5	0.6	0.6	0.9	1.6^∗^	0.8^∗^	1.2^∗^	8.7^∗^	0.7^∗^

*^∗^Significant difference (p < 0.05) between inoculated plants and non-inoculated (NI) controls.*

### Physical Interaction between Plants and Bacteria

**FIGURE 4:**
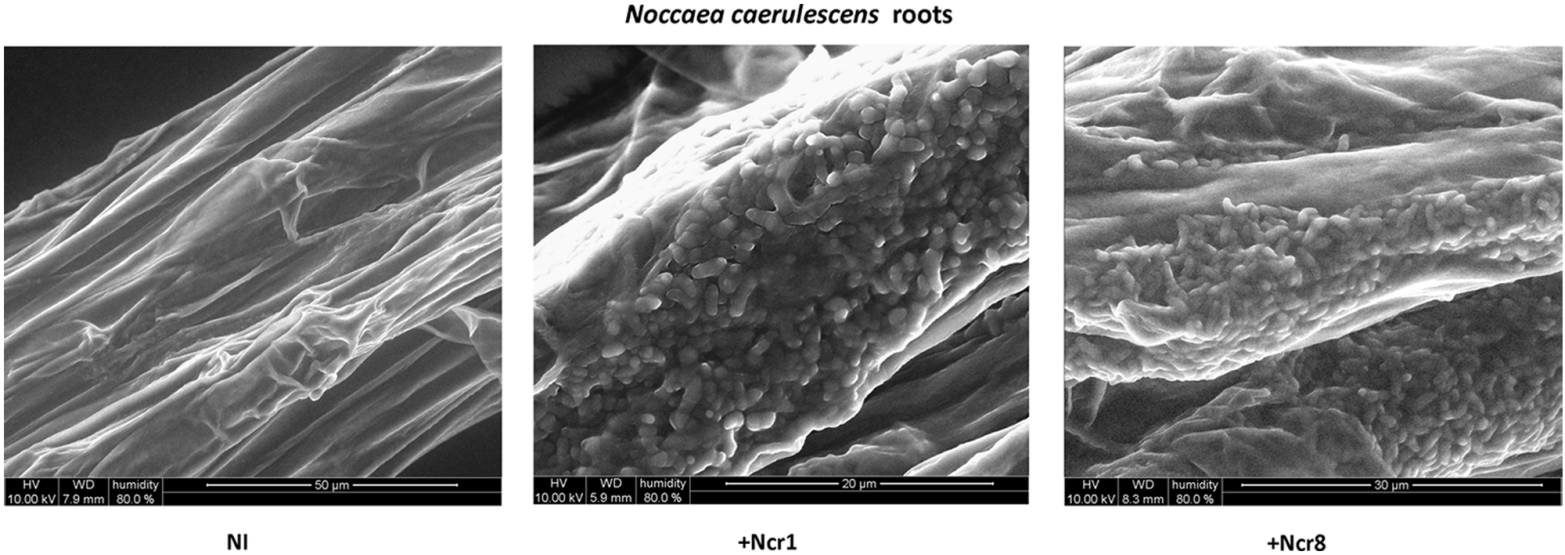
***In vivo* wet-ESEM micrographs of fresh roots from 14-day-old *N. caerulescens* seedlings inoculated with Ncr-1 and Ncr-8 PGPE strains and non-inoculated (NI)**.

**FIGURE 5:**
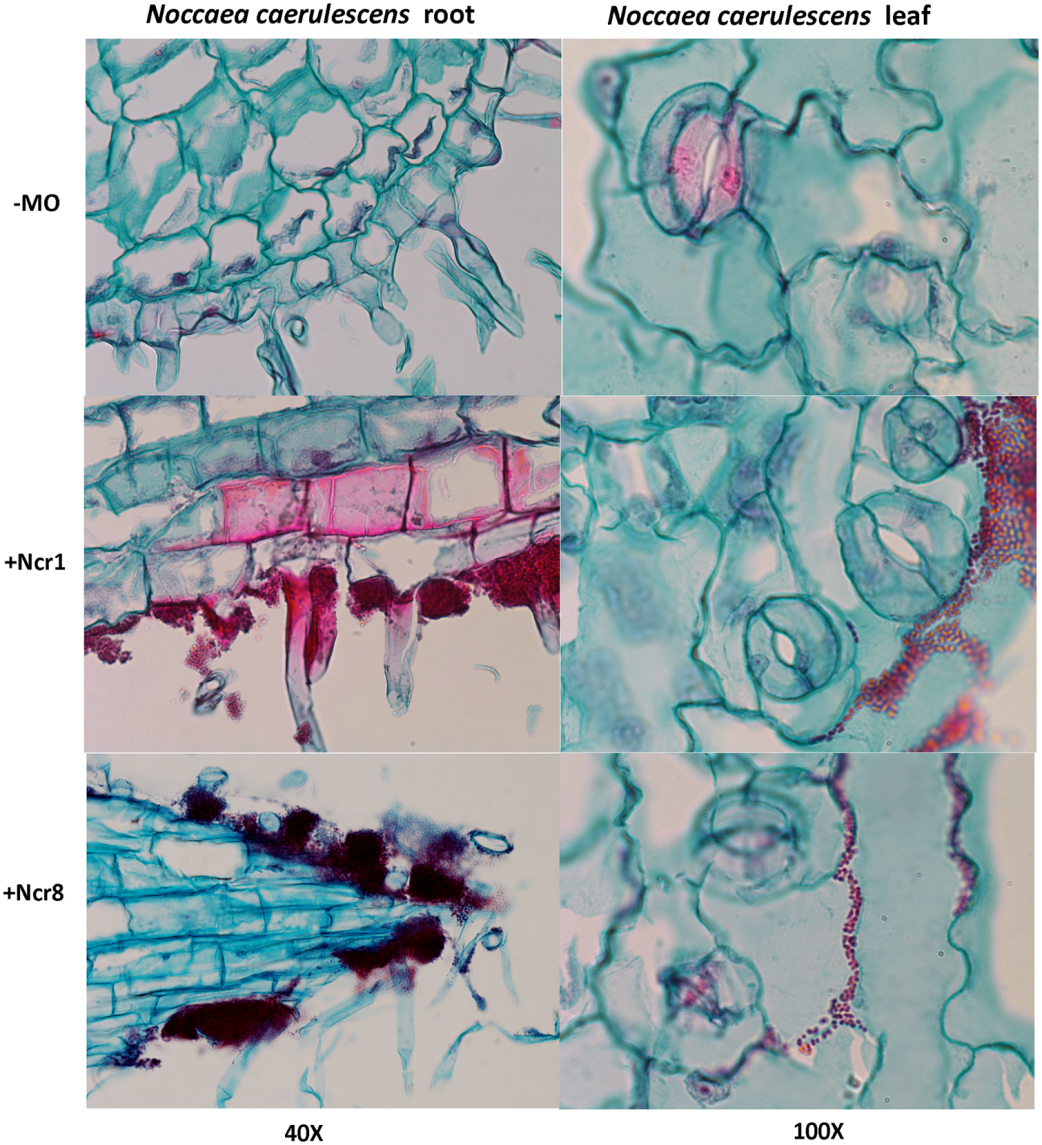
**Histological analysis of longitudinal root **(left)** and leaf **(right)** sections of 14-day-old *N. caerulescens* seedlings inoculated with Ncr-1 and Ncr-8 PGPE strains and non-inoculated (NI).** Bacterial cells were highlighted with safranin and plant cells with fast green staining.

An important aspect to consider in root–microbe interactions is the possibility of tracking bacterial growth and plant tissue colonization (Visioli et al., 2015). Some authors have recently followed bacterial colonization after a certain time from inoculation by means of classic microbiological methods, with selection of metal-resistant bacteria. The bacteria are then identified by colony morphology traits, metal-contamination tolerance, and IAA production and ACC-deaminase activity (Ma et al., 2015b). Until now, very few studies have shown physical plant–microbe interactions in the tissues of hyperaccumulator plants. We monitored the colonization and survival of inocula in real environmental conditions by environmental scanning electron *in vivo* microscopy (ESEM). ESEM is a powerful tool which allows observation of biological specimens *in situ* without sample preparation (Stabentheiner et al., 2010). The physical association between the roots of *N. caerulescens* and Ncr-1 and Ncr-8 was analyzed on 14-day-old seedlings from inoculated seeds before the soil experiments were set up. Single Ncr-1 and Ncr-8 strains showed deep colonization in root cavities and deep bacterial root biofilm formation (**Figure 4**). Images obtained after histological staining of 14-day-old *N. carulescens* seedlings inoculated with single Ncr-1 and Ncr-8 bacteria, compared with non-inoculated plants, are shown in **Figure 5**. Both strains adhered closely to the root epidermis and root tips. They were also very abundant on leaf tissues, penetrating the intercellular spaces between epidermal cells and crowding particularly round the stomata complexes. This finding is noteworthy, because epidermal cells are the primary sites of Ni accumulation in this hyperaccumulator species, although clear exclusion of Ni from guard cells was also recently reported by Mattarozzi et al. (2015).

### Ncr-1 and Ncr-8 Co-Inoculum Enhances *Arabidopsis* Root Growth and Tolerance to Ni

**FIGURE 6:**
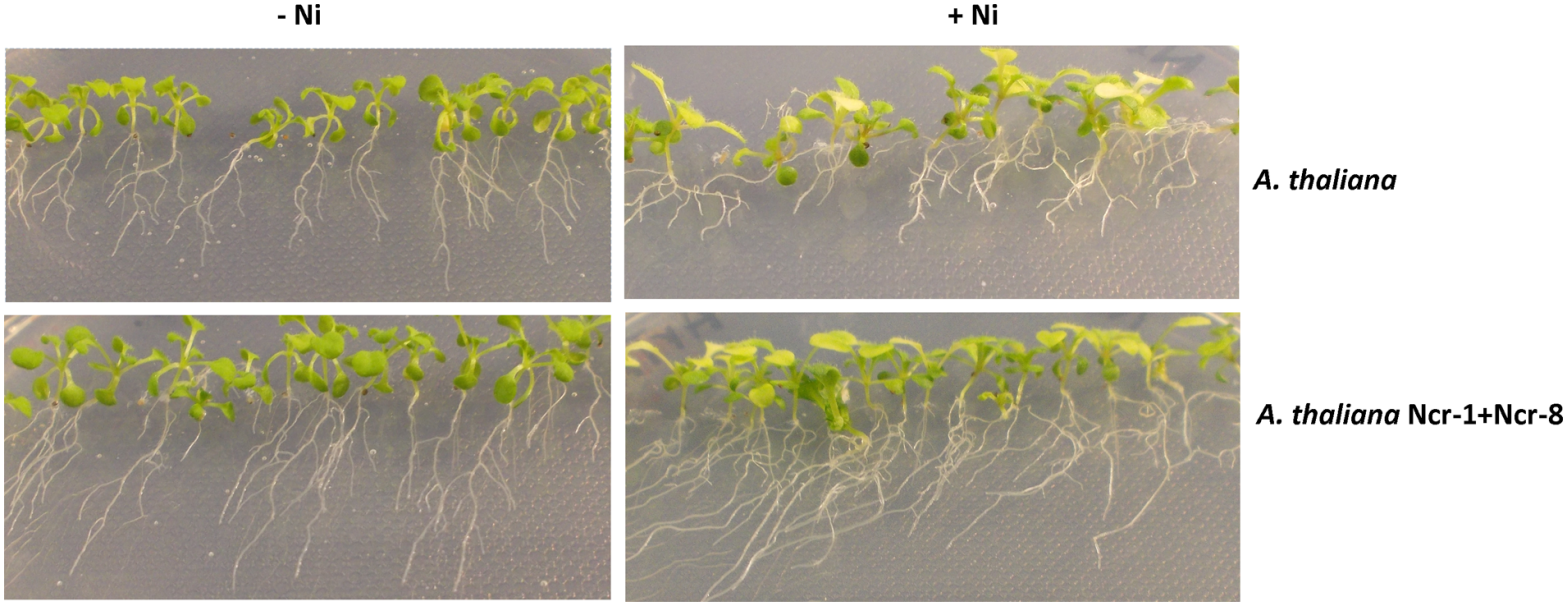
**Seven-day-old *Arabidopsis thaliana* seedlings, non-inoculated (NI) or co-inoculated with Ncr-1 and Ncr-8 PGPE strains growing in MS 1× medium, with or without 40 μM NiSO_4_**.

The effectiveness of the combination of Ncr-1 and Ncr-8 was also tested in the non-hyperaccumulator *A. thaliana.* **Figure 6** shows non-inoculated vs. Ncr-1 + Ncr-8 co-inoculated 7-day-old seedlings. The treated plants revealed enhanced root and shoot growth both with (40 mM NiSO_4_) and without Ni contamination (**Table 7**). In addition, when the growing medium was contaminated by Ni, the inoculated plants clearly showed fewer symptoms of phytoxicity than controls, with a ∼50% increase in root length and an ∼30% increase in plant biomass compared with non-inoculated controls. Although bacteria led to reduced Ni concentration in plant tissues, the balance of metal removal was still better than that of controls (*p* < 0.05; **Table 7**).

**Table 7:** Root length, fresh plant weight, and Ni content (±SE, *n* = 3) in 7-day-old *Arabidopsis thaliana* seedlings grown *in vitro* under 0 or 40 μM NiSO_4_, with or without inoculation.

*A. thaliana*	Root lenght (mm)	Bacterial effect	FW (mg plant^-1^)	Bacterial effect	Ni (mg kg^-1^)	Bacterial effect
NI	3.24 ± 0.2b		45 ± 0.5b			
Ncr-1 + Ncr-8	4.12 ± 0.4a	+27%	52 ± 1.2a	+16%		
NI + Ni	2.94 ± 0.3c		42 ± 2.2b		73 ± 4.3	
Ncr-1 + Ncr-8 + Ni	4.44 ± 0.2a	+51%	55 ± 1.5a	+31%	60 ± 5.3	-17.8%

*Data of each plant batch (three replicates) derived from three plants. Values with different letters: significantly different among treatments (p < 0.05). NI, non-inoculated plants; FW, fresh plant weight.*

Although the role of PGPR in promoting growth and Ni uptake in hyperaccumulators has often been reported (Visioli et al., 2015), the protective effect against Ni toxicity exerted by metal-resistant PGPR or PGPE in non-accumulator biomass species has rarely been documented. For instance, Someya et al. (2007) demonstrated that the *Pseudomonas putida* ARB86 strain isolated from a Ni-contaminated soil could increase *Arabidopsis* plant growth and reduce Ni influx. The PGPR *Kluyvera ascorbata* strain, isolated from a Ni-Cu mining area, protected canola and tomato from Ni toxicity, mainly by stimulating root growth, but did not hamper Ni accumulation by the plant (Burd et al., 1998). The positive role played by these bacteria appear to be similar to those of our Ncr-1 and Ncr-8 strains in the presence of Ni, exerting a growth-promoting effect in roots and probably reducing plant stress thanks to reduced ethylene production (see **Table 1**). In our case, the Ni concentration in *Arabidopsis* tissues was reduced as a consequence of bacterial inoculation, probably because of metal dilution in a more elevated biomass or reduction in uptake. The absolute difference between inoculated and control plants of *A. thaliana* was minimal when compared with that of *N. caerulescens*, and *A. thaliana* maintained the characteristics of a non-hyperaccumulator, with a much lower order of magnitude for Ni accumulation (mg kg^-1^ vs. g kg^-1^).

Extensive research is necessary to examine the possible influence of PGPR and PGPE inoculation on changes of speciation of toxic metals in the rhizosphere and to ascertain whether such changes can alter the accumulation and distribution in plant organs of heavy metals in hyperaccumulator and non-hyperaccumulator plants.

## Conclusion

Highly Ni-polluted serpentine soils are populated by a wide range of bacterial species and strains which play an active role in plant adaptations to extreme soil conditions. Culturable root endophytic bacteria represent only the evaluable part of the community of rhizosphere microorganisms, and the involvement of viable but not cultivable (VBNC) bacteria cannot be excluded.

In this paper, we demonstrate that individual PGPE culturable bacteria are ineffective in plant growth and Ni accumulation enhancement, although they were selected for their high Ni resistance. Among selected strains, those belonging to the *Arthrobacter* and *Microbacterium* genera alone led to better plant performance, but revealed a synergistic effect when associated as seed inocula in *N. caerulescens*. Very probably, co-inoculation of various PGPE bacteria partially mimicks the natural conditions of serpentine soils, in which multiple microorganism interactions occur, helping plants to cope with the toxic effects of heavy metals. Co-inoculation can also improve the phytoextraction of various metals at the same time, as we found for Ni, Co, and Cu, which indicates the possibility of exporting the technology to multiple metal contaminated sites. For these purposes, bacterial strain selection is recommended to be based on metal resistance and IAA over productivity, but with particular attention to ACC-deaminase activity, which reduces plant stress, aging and senescence.

Attempts to extend the technology to non-hyperaccumulator plants led to positive results in terms of root and shoot growth in *A. thaliana*, although with low tissue Ni concentration, not comparable with the results from *N. caerulescens*. However, our isolates can contribute to the creation of a “phytoremediating bacteria database,” to be tested on high biomass-yielding plant species under multiple metal-contaminated sites for phytoextraction purposes.

## Conflict of Interest Statement

The authors declare that the research was conducted in the absence of any commercial or financial relationships that could be construed as a potential conflict of interest.
